# Investigating Gray and White Matter Structural Substrates of Sex Differences in the Narrative Abilities of Healthy Adults

**DOI:** 10.3389/fnins.2019.01424

**Published:** 2020-01-29

**Authors:** Georgia Angelopoulou, Erin L. Meier, Dimitrios Kasselimis, Yue Pan, Dimitrios Tsolakopoulos, George Velonakis, Efstratios Karavasilis, Nikolaos L. Kelekis, Dionysios Goutsos, Constantin Potagas, Swathi Kiran

**Affiliations:** ^1^Neuropsychology and Language Disorders Unit, 1st Department of Neurology, Eginition Hospital, National and Kapodistrian University of Athens, Athens, Greece; ^2^Sargent College of Health & Rehabilitation Sciences, Boston University, Boston, MA, United States; ^3^Department of Neurology, Johns Hopkins University School of Medicine, Baltimore, MD, United States; ^4^Division of Psychiatry and Behavioral Sciences, School of Medicine, University of Crete, Heraklion, Greece; ^5^2nd Department of Radiology, General University Hospital “Attikon”, Medical School, National and Kapodistrian University of Athens, Athens, Greece; ^6^Department of Linguistics, School of Philosophy, National and Kapodistrian University of Athens, Athens, Greece

**Keywords:** speech rate, narration, gray matter, white matter, sex differences

## Abstract

Linguistic aspects of narration have been investigated in healthy populations, in a wide variety of languages and speech genres with very different results. There is some evidence indicating that linguistic elements, such as speech rate (i.e., the measure indicating the amount of speech produced in a certain time period), mean length of utterance (MLU) (i.e., the index reflecting sentence grammatical structure), frequency of nouns and verbs, might be affected by non-linguistic factors such as sex. On the other hand, despite the existence of neuroimaging evidence of structural differences between males and females, it is yet unknown how such differences could explain between-sex disparities in linguistic abilities in natural speech contexts. To date, no study has evaluated discourse production elements in relation to sex differences and their neural correlates in terms of brain structure, a topic that could provide unique insights on the relationship between language and the brain. The aim of the present study was to determine sex differences in narrative skills in healthy adults and to investigate white and gray matter structural correlates of linguistic skills in each group. Twenty-seven male and 30 female (*N* = 57) right-handed, neurologically intact, monolingual Greek speakers, matched for age and years of education, participated. Narrations of a personal medical event were elicited. Linguistic elements of speech rate (words per minute), MLUs, frequency of nouns and verbs were calculated for each speech sample, by two independent raters. Structural 3D T1 images were segmented and parcellated using FreeSurfer and whole-brain between-sex differences in cortical thickness, cortical volume and surface area, were obtained. Between-group differences in white matter diffusion tensor scalars were examined via Tract-Based Spatial-Statistics and whole-brain tractography and automated tract delineation using Automated Fiber Quantification. Speech rate and noun frequency were significantly lower for men, while verb frequency was significantly higher for women, but no differences were identified for MLU. Regarding cortical measures, males demonstrated increased volume, surface area and cortical thickness in several bilateral regions, while no voxel-wise or tractography-based between-group differences in white matter metrics were observed. Regarding the relationship between sex and speech variables, hierarchical regression analyses showed that the superior/middle frontal cluster in surface area may serve as a significant predictor of speech rate variance, but only in females. We discuss several possible interpretations of how sex-related speech abilities could be represented differently in men and women in gray matter structures within the broad language network.

## Introduction

Whether sex differences in cognition exist is a question of high scientific interest across different scientific subfields of biology, psychology, and social sciences (Choleris et al., 2018). The general notion of discrepancies between males and females in specific cognitive domains gradually evolved starting from the early 1900s and eventually was established around the middle of the 20th century (see Hyde, 2016, for a review and a brief historical overview): in a nutshell, males are thought to outperform the opposite sex when it comes to spatial and mathematical tasks, while females are supposed to exhibit superior verbal skills. Despite a significant number of studies supporting this sex-dependent dichotomy in relation to cognitive abilities, reviews and meta-analyses highlight the need to further scrutinize the aforementioned sex asymmetries in cognition (Wallentin, 2009; Hyde, 2016).

The most commonly investigated verbal skills to demonstrate possible sex differences in healthy children and adults are verbal fluency (Tombaugh et al., 1999; Weiss et al., 2003), verbal memory (Bleecker et al., 1988; Vakil and Blachstein, 1997; Lewin et al., 2001) and vocabulary, while between-sex differences have also been noted for reading comprehension, essay-length written production and speech production (Hyde and Linn, 1988). However, limitations such as age range and different sampling methods render the generalization of these results problematic. On top of that, meta-analyses indicate that effect sizes for between-gender differences appear relatively small, thus complicating the issue even more (see Hyde and Linn, 1988 for a meta-analysis; Herlitz and Rehnman, 2008 for a discussion of sex differences in episodic memory and Wallentin, 2009 for a critical review on sex differences in language).

Data for between-sex comparisons in Greek are mainly derived from normative studies. In their study about verbal fluency, Kosmidis et al. (2004) found that women perform significantly better in a lexical-semantic condition compared to phonological condition and specifically in total number of uttered words. Regarding verbal comprehension, no sex discrepancy in performance was found in a study that included auditory comprehension task, which was administered to a large cohort of healthy participants (Simos et al., 2014), similar results were obtained in another study focused on a verbal learning task (Constantinidou et al., 2014). Another study reported significantly higher scores for men in receptive vocabulary-regardless of age, years of formal schooling and naming ability-but similar performance across sexes for expressive vocabulary (Simos et al., 2011). Even though these studies include large samples with appropriate demographic characteristics such as a wide range of age and years of formal schooling, adequate representation of both sexes and recruitment from several geographic areas of Greece, making generalization of results is plausible, they do not provide any data on speech narratives.

Sex differences with regard to language have been studied by several scholars, yet data focused on discourse characteristics of different narration genres are scant. It is noteworthy that while other demographic factors, like age, have been thoroughly investigated in connected speech (see Mortensen et al., 2006, for a review on the effects of age on speech production), little is known about differences between males and females since most of these studies ignore sex as a possible factor contributing to variability of linguistic indices and different patterns of speech output characteristics.

While studies focusing on sex differences in several fluency measures during text level oral speech production are limited, those that do exist present contradictory findings. Ardila and Rosselli (1996) investigated sex and education effects on total number of words, ratio of nouns, verbs, adjectives and connectors during a picture description task in a large cohort of 180 Spanish-speaking healthy individuals. Their results showed that only total number of words was significantly different between males and females; however, that difference was modulated by age, since it appeared only in the third age group (age range: 51–65), while no differences were found for the other two age groups (16–30 and 31–50 years of age). The authors attributed the observed sex differences to possible contributions of biological and social factors, acknowledging the difficulty of pinpointing the exact cause of the differences. The challenge of identifying the underlying nature of between-sex differences in narrative production could be due to the complex nature of narration tasks. For example, various cognitive resources are needed to successfully describe a picture that contains multiple visual elements, each of which corresponds to different phonological and semantic representations.

Mackenzie (2000) compared two genres of connected speech in order to investigate possible effects of age, education, and gender. Speech samples were acquired in a conversational interaction condition and during picture description from a cohort of 189 neurologically intact adults. Specific variables as initiation, topic maintenance, verbosity, turn taking and reference were annotated for conversations and content, length, efficiency and the inclusion of extraneous information for picture description. Although a trend for better performance by women was observed regarding content in both tasks, results failed to reach significance. A similar trend was presented by Leeper and Culatta (1995) for different language variables. They investigated the effect of age and sex on fluency markers such as total number of words and total number of dysfluencies, as interjections, revisions and hesitations, derived from two narration tasks, a picture description and a free monolog on a topic of each subject’s choice and a reading task, from a sample of 98 healthy participants separated in four age groups with an age range from 55 to 85+ years old. Results indicated a trend of increased dysfluencies in older males for all tasks, however significant differences were observed only in reading ability. A cross-linguistic study by Juncos-Rabadán (1996) is in accordance with the above findings, providing evidence against sex differences. The study assessed elderly healthy speakers’ ability to retell stories, by measuring the following speech variables: story structure and quality, tangential and descriptive sentences, cohesion links and place deixis. While results indicated a negative effect of age and a positive effect of education regardless of language, the effect of sex was not significant.

Wardle et al. (2011) found that females demonstrate higher speech rate compared to males on two different free narration tasks. More specifically, they investigated the effect of IQ, personality traits, and several demographic factors on performance in five verbal tasks, including a verbal fluency task and different forms of connected speech, as dialogue, monolog, interpersonal and unprompted speech, in 51 (32 females) English-speaking young adults, with age ranging from 18 to 35 years. Variables extracted from narrative tasks included word count, narration duration and speech rate. Results indicated that females spoke faster in the monolog and interpersonal conditions, while no significant differences between sexes were observed for the other tasks. Verhoeven et al. (2004) found the opposite result in a sample of healthy Dutch speakers. More specifically, they measured speech rate in 160 Dutch native speakers from different areas of the Netherlands and Belgium, aiming to investigate possible effects of different areas’ citizenship on spontaneous speech. Results indicated that males have higher fluency values than females.

Finally, the few corpus studies of gender-related differences in English suggest that women consistently use fewer nouns than men both in present-day language (Rayson et al., 1997; Argamon et al., 2003) and in the history of the language (Saily et al., 2011). However, there are no respective studies in Greek.

In sum, there is lack of consensus in the sparse research evidence regarding sex effect in narrative ability. This could be due to the adoption of different methodological approaches. An important factor that should be taken under consideration in the attempt to summarize and synthesize the available findings is the selection of different narration genres (as picture description, story retelling, narration of personal events, procedural discourse) across studies. Each type provides unique information about a speaker’s ability to organize and produce speech, as it incorporates different cognitive demands (Bliss and McCabe, 2006), and therefore results across different speech genres are not necessarily comparable. Overall, the variability of elicitation tasks, linguistic indices, and sample characteristics across the relevant studies may explain the observed discrepancy and subsequent lack of consensus across languages. Moreover, to our knowledge, evidence from previous studies in Greek aimed at identifying sex differences in connected speech do not exist.

Furthermore, the structural instantiation in the brain of potential between-sex differences in narrative indices has not been thoroughly investigated. Various attempts have been made to investigate the structural and functional differences between males and females, yet consistency in between-sex brain differences remains terra incognita. For example, consistent evidence pointing to systematic patterns of morphological differences in brain is still vague, while it also remains unclear how these between-sex differences actually manifest (see for a review, Ruigrok et al., 2014). Nevertheless, recent meta-analyses highlight the need to include sex as a contributing factor in statistical models involving brain-related data (see for a review, Sacher et al., 2013).

Several studies have demonstrated differences between sexes in gray matter indices, as volume and thickness. For example, Ritchie et al. (2018) conducted one of the largest studies (including 5126 participants) aiming to investigate the multimodal nature of sex differences in adult human brain structural and functional organization. Their findings are in favor of differences existing in both structure and function of the brain. More specifically, males presented greater volume and surface area in cortical areas including extensive bilateral parietal regions for volume and bilateral temporal for area, even when controlling for total intracranial volume – which is greater in men. Smaller-scale studies seem to be in accordance with the general notion of inter-sex differences in brain structure. In a study of 465 neurologically intact adults, Good et al. (2001) found that males exhibited significantly higher gray matter volume in the majority of brain regions, yet they also found some clusters in which women had higher volumes than men. Similarly, Carne et al. (2006) found that males demonstrated greater cortical volumes in the frontal, temporal and occipital lobes of left hemisphere while females had greater gray matter volume in left parietal lobe. Chen et al. (2007) examined a group of healthy individuals within a rather restricted age range (44–48 years old) and found that in general, men had larger brain volumes in left inferior temporal gyrus, right occipital lingual gyrus, right middle temporal gyrus, whereas women exhibited higher gray matter volume in dorsal anterior, posterior and ventral cingulate cortices, as well as right inferior parietal lobule.

Diffusion data from other studies also seem to confirm the existence of brain differences between sexes (Sacher et al., 2013). In most of the studies it has been suggested that women have higher fractional anisotropy values (see for example Kanaan et al., 2012, 2014; Dunst et al., 2014). In contrast, though, Ritchie et al. (2018) noted that men had higher fractional anisotropy values, in 18 out of 22 tracts that were examined, but women demonstrated higher indices of tract complexity in most of the tracts. Women also had a greater proportion of gray matter compared to white matter volume, but this finding has also been questioned as there is evidence indicating that women have reduced gray and white matter, compared to men (see Sacher et al., 2013, for a review). It has been suggested that the more robust structure of the corpus callosum in men compared to women (as indexed by fractional anisotropy) can explain between-sex differences in other brain areas, but this idea has also been criticized (Westerhausen et al., 2011).

Based on the literature reviewed above, sex-related differences in brain structure often include cortical perisylvian regions and white matter tracts implicated in language processing (see Wallentin, 2009, for a critical review), but how such anatomical differences are related to between-sex differences in language processing remains relatively unknown. Functional studies investigating sex differences in language lateralization also present contradictory results (see for a discussion: Kansaku and Kitazawa, 2001). For example, there are some studies suggesting that males exhibit unilateral (i.e., predominantly left), while females show bilateral activation of anterior perisylvian areas during phonological (Shaywitz et al., 1995) and grammatical and reading tasks (Jaeger et al., 1998). Similar inter-sex differences in activation of posterior perisylvian areas have been also found during a listening task (Kansaku et al., 2000). On the other hand, there are studies and metanalyses presenting null results (Sommer et al., 2004, 2008). Such controversies have been attributed to several factors, including the different measurement processes and methodologies applied, the control of nuisance variables, the nature of the task used, as well as sample size (for a review see also Wallentin, 2009).

The present study aims to examine possible differences between sexes with regard to narrative abilities, and to further correlate linguistic indices derived from the acquired speech samples with between-sex differences in gray and white matter. Most of the studies described above discuss whether there are differences between males and females with regard to brain structure *or* linguistic behavior. However, to the best of our knowledge, there is no study thus far attempting to integrate anatomical and language data, in order to provide indications about possible associations of inter-sex differences with regard to *both* brain structure and linguistic behavior. Given the fact that findings related to sex differences with regard to narrative indices and possible neural correlates are scarce, inconclusive or absent, and taking into consideration that spontaneous language production is central to the assessment of language disorders (Bliss and McCabe, 2006), and is of higher ecological validity (for a discussion, see Angelopoulou et al., 2018), we argue that this study will contribute to the understanding of the differences in linguistic behavior between men and women.

## Methodology

### Participants

For the purpose of this study, we recruited 57 individuals, including twenty-seven males, 19–64 years old (mean: 44.11, *SD*: 13.8) and 30 females, 21–65 years old (mean: 44.57, *SD*: 10.9). All participants were right-handed, monolingual Greek speakers. The two groups were matched for age and years of education, with no history of neurological or psychiatric disorders (see Table 1 for demographics).

**TABLE 1:** Demographic characteristics of the two groups.

	**Males**		**Females**		
	**Range**	**Mean (*SD*)**	**Range**	**Mean (*SD*)**	p*
Age (years)	19–64	44.11 (13.8)	21–65	44.57 (10.9)	0.457
Education (years)	12–25	16.00 (3.9)	9–22	15.30 (3.1)	0.890

*Independent samples *t*-test.

All participants were sampled from the project “Investigation of cortical surface patterns and their relation with speech metrics and performance in neuropsychological assessment in healthy participants,” conducted at Eginition Hospital in Athens, School of Medicine, Greece (research protocol approval ID: ΩOΞΛ46Ψ8N2−7PN, July 2017). Informed consent was obtained from all participants prior to participation according to Ethics Committee of Eginition Hospital.

### Narration Sample Analysis

Participants were asked to describe a medical event of their own or of someone related to them, as a recount of a past event (see Armstrong, 2000 for a similar narration genre). There were no restrictions in narration time. The speech samples were recorded and then orthographically transcribed following the basic conventions of discourse analysis in Greek (Georgakopoulou and Goutsos, 2011) and the coding conventions of the Corpus of Greek Aphasic Speech (Goutsos et al., 2011). Inter-rater agreement was measured by comparing orthographical transcriptions from three researchers, two from the Department of Linguistics and one from the Department of Neurology. Each researcher first individually transcribed the recordings before comparing all sets of data and resolving any discrepancies until they reached 100% agreement. Linguistic elements of speech rate (words per minute), mean length of utterances (MLU), number of function words, frequency of nouns (as number of nouns per 100 words) and number frequency of verbs (as number of verbs per 100 words), were calculated according to the speech annotation method *Quantitative Production Analysis* (QPA), proposed by Saffran et al. (1989) and adapted for Greek by Varkanitsa (2012). For the speech rate calculation, total duration of the audio files was used for each participant, while for all the other linguistic metrics, only the first 100 words were analyzed based on the QPA updated methodology as proposed by Rochon et al. (2000) and has been implemented in other studies (see for example Efthymiopoulou et al., 2017). In order to calculate MLU, speech samples were segmented into utterances using primarily semantic, syntactic and intonational criteria in accordance with speech annotation methodology for Greek proposed by Varkanitsa (2012), which we have previously implemented in patients with aphasia and healthy speakers (Angelopoulou et al., 2018).

### Magnetic Resonance Imaging: Acquisition Protocol

3D T1-weighted, 30-directional DTI protocol and T2-FLAIR were acquired for all participants on a 3T Philips Achieva-Tx MR scanner (Philips, Best, Netherlands), equipped with an eight-channel head coil.

T1-weighted sequence had the following parameters: repetition time = 9.90 ms; echo time = 3.69 ms; flip angle = 70°; 170 contiguous 1 mm slices; field of view = 250 × 250 mm; matrix size = 256 × 256, voxel size = 1 × 1 × 1 mm^3^; slice thickness = 1 mm.

DTI protocol acquisition included an axial single-shot spin-echo echo-planar imaging (EPI) sequence with 30 diffusion encoding directions and the following parameters: repetition time (TR): 7299 ms; echo time (TE): 68 ms; flip angle: 90°; field of view (FOV): 256 × 256 mm; acquisition voxel size: 2 × 2 × 2 mm. The acquisition consisted of 70 slices and the scan time was 8 min 40 s.

T2-FLAIR were included to the acquisition protocol, to exclude participants with cerebrovascular disease. All T2-FLAIR were examined by an experienced neuroradiologist. None of the included participants exhibited any indicators of cerebrovascular disease (as lacunes, leukoaraiosis) or cortical atrophy not compatible with age.

### Magnetic Resonance Imaging: Processing and Between-Sex Statistical Tests

#### Surface-Based Analysis

Whole brain cortical reconstruction of T1 MR images was obtained using the standard pipeline of FreeSurfer 6.0.0^1^ (Dale et al., 1999; Fischl et al., 1999; Fischl and Dale, 2000). This process included motion correction by linear transformation, accurate skull stripping, and cortical segmentation based on identification of gray/white matter boundaries in native space. All participants’ images were registered to the common surface space (i.e., the fsaverage atlas) and subsequently smoothed with a Gaussian kernel of FWHM 10 mm. Each hemisphere was modeled separately. Cortical thickness was calculated as the closest distance from the gray/white boundary to the gray/CSF boundary at each vertex on the tessellated surface (Fischl and Dale, 2000).

Whole brain differences in the measurements of surface area (SA), cortical thickness (CTh), and gray matter volume (GMV) were examined for both cerebral hemispheres with separate vertex by vertex general linear models (GLMs), in order to identify differences between sexes in all brain metrics. Age was included as nuisance variable in all models. Total intracranial volume (TIV) was used as a nuisance variable for between-group designs, for volume and surface area measurements, as previously suggested by Yoo et al. (2016). However, for cortical thickness analyses, no covariates were used, as suggested by previous researchers (e.g., Westman et al., 2013). Monte Carlo simulations were used to correct all vertex-wise results at an individual vertex level of *p* < 0.05 (Hagler et al., 2006; see also Johns et al., 2018).

#### Diffusion Images Analysis

Images were processed using the FMRIB Software Library (FSL) software package 5.0.9 (FMRIB, Oxford, United Kingdom)^2^. The standard preprocessing pipeline was implemented using the FMRIB Diffusion Toolbox (FDT). In brief, preprocessing steps included brain extraction, eddy current correction for motion artifacts, and calculation of the diffusion tensor, from which diffusion scalar maps (i.e., axial diffusivity [AD], fractional anisotropy [FA], mean diffusivity [MD] and radial diffusivity [RD]) were derived. Top-up could not be employed to correct for susceptibility artifacts because diffusion data were acquired from only one phase encoding direction.

Further voxelwise statistical analysis of the fractional anisotropy (FA) maps was implemented following the standard TBSS pipeline (Smith et al., 2006) in FSL (Smith et al., 2004) (see for a detailed description, Lövdén et al., 2013). Specifically, images alignment was done through non-linear registration to the FMRIB58_FA standard-space image and the skeletonized mean FA image was created from the aligned images and thinned in order to provide the core of all tracts common to the group. A threshold FA value of 0.2 was applied to the mean FA skeleton map to mitigate partial volume effects of white matter voxels containing gray matter or cerebrospinal fluid (CSF). Individual skeleton images were then created by projecting all participants’ aligned FA images to the mean FA skeleton map. The same procedure was carried out for non-FA scalars (i.e., AD, RD, and MD). In order to test between-group differences in diffusion metrics, we conducted a whole-brain voxel-wise statistical analysis of these skeletonized images through a permutation-based inference of 5000 permutations, correcting with the threshold-free cluster enhancement (TFCE) method [*p* < 0.05 (family-wise error [FWE] corrected)] (Nichols and Holmes, 2002). Age was included as nuisance variables in all TBSS analyses.

Voxel-based techniques such as TBSS cannot ensure that any voxel—especially at tract extremes—corresponds to the same tract location across individuals within a sample. As an alternative, white matter tractography involves using an individual’s own anatomy to “grow” and subsequently measure white matter fascicles *in vivo* and is considered to be a more accurate measure of tract characteristics than voxel-based analyses. Therefore, as a follow-up to TBSS, we conducted whole-brain tractography using the default parameters set in the Automated Fiber Quantification Matlab-based software (AFQ; Yeatman et al., 2012). AFQ has been used in both healthy individuals (e.g., Johnson et al., 2014; Deng et al., 2017) and clinical populations, including individuals with developmental disorders (e.g., Libero et al., 2016; Lin et al., 2018), psychiatric conditions (e.g., Sacchet et al., 2014; Deng et al., 2018) and acquired neurological disorders (e.g., Keller et al., 2017; Main et al., 2017; Sarica et al., 2017; Goodrich-Hunsaker et al., 2018; Zhang et al., 2018). The AFQ procedure is described in detail elsewhere (Yeatman et al., 2012). In brief, DTI data preprocessed in FSL (see above) were first transformed into a useable file format for AFQ. Within AFQ, first, whole-brain fiber tractography is performed for each individual. AFQ estimates tract fibers through a deterministic streamline algorithm using a 1 mm fixed-step size along the principle diffusion axes between seed points within white matter masks representing the ends of each tract. Streamline delineation is terminated if FA values are less than 0.20 and the path angle between steps is greater than 30°. Second, a waypoint region of interest (ROI) technique based off of Wakana et al. (2007) is used to segment 20 tracts of interest, including the corpus callosum forceps major and minor and the left and right inferior fronto-occipital, inferior longitudinal, superior longitudinal, uncinate, and arcuate fasciculi; thalamic radiations; corticospinal tract; and cingulum (split into cingulate and hippocampal sections). Waypoint ROIs within the AFQ software for each tract are warped from MNI space into each subject’s diffusion space, and streamlines that intersect the ROIs are identified as belonging to a given tract. Third, fiber tracts are further refined through an iterative cleaning process. First, each candidate fiber within a given pathway is compared to probability white matter tract maps from Hua et al. (2008). Next, the position of each fiber is determined by dissecting the tract into 100 equidistant nodes between the two waypoints and considering the spread of fibers in 3D space at each node; the tract core represents the mean of each fibers at each node. Tract cleaning removes streamlines that are more than four standard deviations from the mean tract length and five standard deviations in distance from the tract core according to its Mahalanobis distance. This process is iterated until no further fiber outliers remain. Finally, diffusion metrics (i.e., mean AD, FA, MD, and RD) are calculated for each of 100 equidistant bins defined along the core of each final tract.

We used *t*-tests to identify bins that significantly differed between males and females in AD, FA, MD, or RD for each tract. To correct for multiple comparisons, we utilized a script available within AFQ which implements non-parametric permutation tests similar to Nichols and Holmes (2002). This procedure resulted in a family-wise error (FWE)-corrected alpha value and cluster threshold that were used to determine significant between-group differences.

### Relationship Between Anatomical Brain and Language Variables

To examine whether between-sex differences in brain structure are related to narrative speech variables, we implemented the following protocol for each type of imaging analysis (i.e., FreeSurfer, TBSS and AFQ). First, we extracted numerical data for each participant that corresponded to left or right hemisphere clusters (either cortical or subcortical) or tract bins that significantly differed between males and females. Next, we conducted preliminary partial correlation analyses between language variables that differed between men and women (i.e., speech rate, noun frequency, and verb frequency) and the anatomical indices of between-sex differences, controlling for age and years of education. Based on the results derived by the preliminary correlation analyses, hierarchical regression analysis was conducted. The model was run separately for the two genders in order to investigate possible different association patterns between anatomical and language variables in men and women. For the analysis, the first, basic model included age and years of formal schooling as independent variables. The second model included the same demographic factors as control variables; predictor variables of interest included anatomical variables which significantly differed between men and women and were significantly associated with language metrics. Finally, the two regression models within the hierarchical regression analysis were statistically compared using ANOVA. The aforementioned analysis was conducted using SPSS v.22.0.

## Results

### Behavioral Measurements

Independent samples *t*-tests revealed statistically significant differences between men and women’s speech rate [*t*(55) = −2.213, *p* = 0.031], noun frequency [*t*(55) = 2.649, *p* = 0.011], and verb frequency [*t*(55) = −2.848, *p* = 0.006], with women presenting higher speech rate values and verbs’ frequency, while men exhibiting higher nouns’ frequency. No differences were identified for number of utterances, MLUs and function words frequency (see Table 2, for behavioral measurements).

**TABLE 2:** Descriptive statistics for linguistic elements for male and female group.

	**Males**		**Females**		
	**Mean (*SD*)**	**Range (min-max)**	**Mean (*SD*)**	**Range (min-max)**	p*
Speech rate *(words per minute)*	108.70 (19.4)	68.38 (84.71–153.10)	123.20 (28.7)	115.20 (75.68–190.90)	0.031
Number of Utterances	5.33 (1.2)	5.00 (3.00–8.00)	5.70 (1.4)	6.00 (2.00–8.00)	0.311
Mean Length of Utterances *(MLU)*	17.86 (4.9)	18.25 (7.75–26.00)	19.30 (6.8)	40.75 (8.25–49.00)	0.371
Function Words *(percentage of)*	44.91 (6.3)	25.56 (30.00–55.56)	46.83 (5.5)	27.45 (31.31–58.56)	0.225
Verbs *(percentage of)*	17.10 (4.0)	16.46 (9.28–25.74)	20.15 (4.1)	17.15 (10.13–27.27)	0.011
Nouns *(percentage of)*	20.67 (5.4)	24.83 (13.40–38.24)	17.35 (4.0)	16.03 (10.89–26.92)	0.006

*Independent samples *t*-test.

### Between-Sex Comparisons in Structural Variables

#### Gray Matter Analysis

**FIGURE 1:**
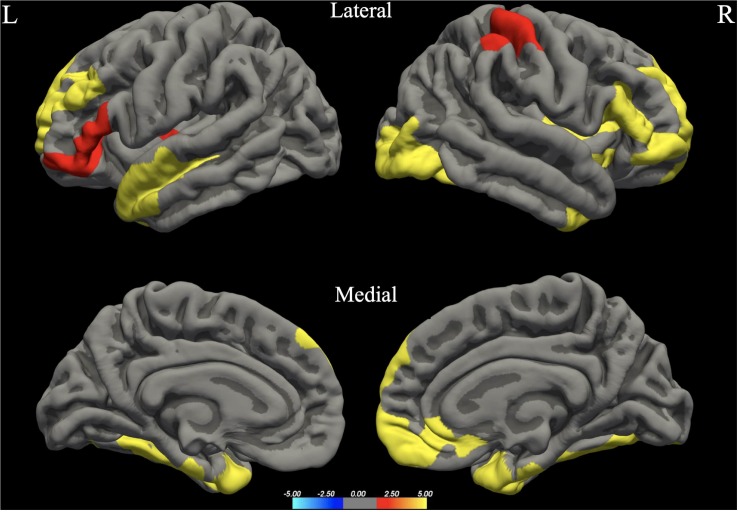
Between-group differences in Surface Area (SA). Warm colors reflect areas where men had higher Surface Area than women. The color bar represents the tenth logarithm of *p*-value. Clusters significant after multiple comparison correction with Monte Carlo simulations (*p* < 0.05).

**FIGURE 2:**
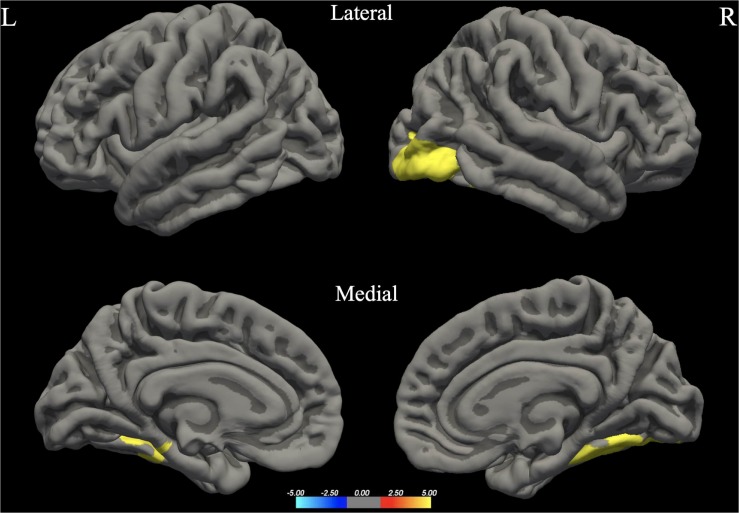
Between-group differences in Gray Matter Volume (GMV). Warm colors reflect areas where men had higher Gray Matter Volume than women. The color bar represents the tenth logarithm of *p*-value. Clusters significant after multiple comparison correction with Monte Carlo simulations (*p* < 0.05).

**FIGURE 3:**
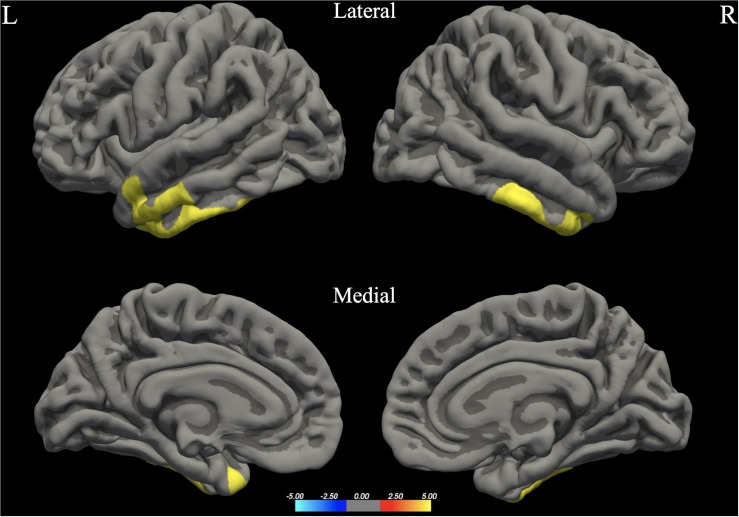
Between-group differences in Cortical Thickness (Cth). Warm colors reflect areas where men had higher Cortical Thickness than women. The color bar represents the tenth logarithm of *p*-value. Clusters significant after multiple comparison correction with Monte Carlo simulations (*p* < 0.05).

The whole brain analysis GLMs for cortical thickness, surface area and gray matter volume demonstrated several significant differences between males and females (Monte Carlo corrected, *p* < 0.05). Specifically, males presented with increased values in surface area, gray matter volume and cortical thickness, bilaterally (see Figures 1–3; see Table 3 for specific clusters).

**TABLE 3:** Significant clusters for male > female group differences in cortical thickness, surface area and gray matter volume.

	**Hemisphere**	Clusters*	**Area (mm^2^)**	***t***	***p***	***x***	***y***	***z***
Gray matter volume	RH	Lateral occipital (also including areas of IOG, IOP, Opole)	3622.25	4.261	0.00020	39.7	–85.2	–13.5
	LH	Fusiform (also including areas of OcctempS and LingS)	993.69	3.749	0.02958	–37.1	–41.4	–22.4
Surface area	RH	Fusiform (also include areas of Ins, IFGtri, IFS, SFG, OcctempS, Opole, IOG, IOP)	13904.14	4.462	0.00020	32.8	2.3	–41.8
		Supramarginal (also including areas of PCG and PCS)	2129.46	2.993	0.00479	57.3	–31.9	44.7
	LH	Superior Temporal Gyrus (also including areas of STS, TP, Plan.Temp, OcctempS, Fus.G.)	4736.60	3.492	0.00020	–47.8	6.6	–25.3
		Superior Frontal Gyrus (also including areas of SFS, MFG, FP)	3470.73	2.972	0.00020	–16.6	56.9	15.2
		Inferior Frontal Gyrus – Pars Triangularis (also including areas of Orb.G, IFGorb, Ins)	2172.73	3.007	0.00220	–35.4	28.2	5.9
Cortical thickness	RH	Inferior Temporal Gyrus (also including areas of TP)	1115.83	3.832	0.00599	42.5	–3.1	–41.7
	LH	Inferior temporal (also including areas of TP and MTG)	2164.34	5.092	0.00020	–41.9	–8.3	–41.0

*Based on Desikan-Killiany Atlas parcellation (2006). Abbreviations of areas included in clusters: IOG, Inferior Occipital Gyrus; IOP, Inferior Occipital Sulcus; Opole, Occipital Pole; OcctempS, Occipito-temporal Sulcus; LingS, Lingual Sulcus; Ins, insula; IFGtri, Inferior Frontal Gyrus - Pars Triangularis; IFS, Inferior Frontal Sulcus; SFG, Superior Frontal Gyrus; PCG, Post Central Gyrus; PCS, Post Central Sulcus; STS, Superior Temporal Sulcus; TP, Temporal Pole; Plan.Temp, Planum Temporale; Fus.G., Fusiform Gyrus; SFS, Superior Frontal Sulcus; MFG, Middle Frontal Gyrus; FP, Frontal Pole; Orb.G, Orbitofrontal Gyrus; IFGorb, Inferior Frontal Gyrus – Pars Orbitalis; MTG, Middle Temporal Gyrus.

#### White Matter Tracts (TBSS and AFQ)

**FIGURE 4:**
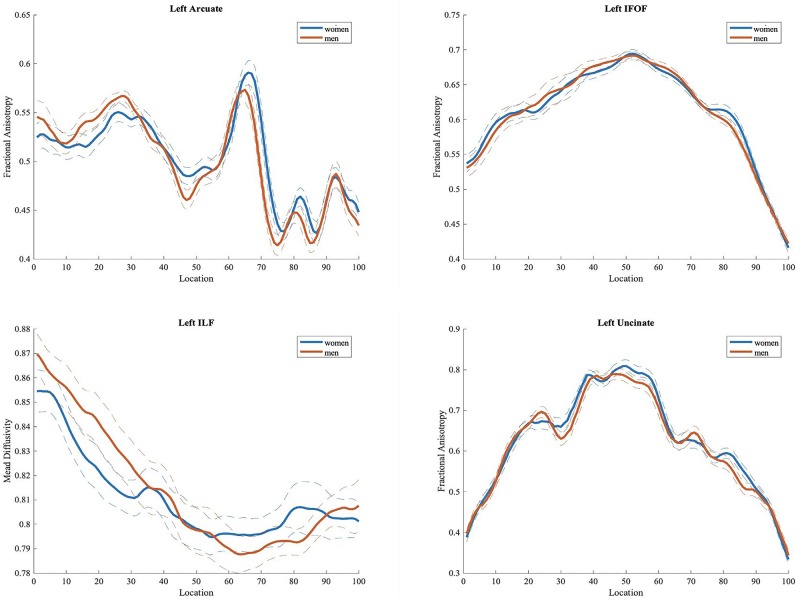
Fractional anisotropy (FA) values in men and women for left hemisphere tracts (arcuate, inferior fronto-occipital, inferior longitudinal and uncinate fasciculi) often implicated in language processing. Plots of mean FA values are reported bin by bin for each group (women in blue and men in orange). Dotted lines represent ± 1 SD. The *x*-axis represents location along the length of each tract core from 1 to 100 equidistant bins. The *y*-axis reflects the subjects’ group mean FA values for each tract.

We did not find any significant difference in FA, MD, RD, and AD values between the two groups, in either tract based spatial statistics or automated tractography (see Figure 4).

#### Relationship Between Anatomical Brain and Language Variables

Because no between-sex differences in white matter metrics were found, we did not conduct follow-up analyses investigating relationships between language variables and variables extracted from the TBSS or AFQ analyses.

In contrast, as shown in Table 3, we identified several cortical clusters that significantly differed between sexes, in both hemispheres. Thus, we extracted individual-level data (either cortical thickness, gray matter volume or surface area, depending on the area) from each cluster in Table 3, and we conducted partial correlations (controlling for demographic variables) between these metrics of both hemispheres and language variables that significantly differed between men and women (i.e., speech rate, verb frequency, and noun frequency), separately for each sex group. As shown in Table 4, no significant relationships were found between cortical clusters and percentage of nouns or verbs. On the other hand, we found a significant association between speech rate and surface area of the superior frontal gyrus cluster (*r* = 0.508, *p* = 0.006), for females’ group only. No correlations appeared to be significant between any speech metric and clusters of the right hemisphere, for any group. To further explore the significant association between speech rate and surface area of the superior frontal gyrus cluster, two sets of hierarchical regression analyses were constructed, one for each group, where in the dependent variable was speech rate and the independent anatomical predictor in the complex regression models also included surface area of the superior frontal gyrus.

**TABLE 4:** Partial correlations between speech variables and cortex clusters, for males and females (nuisance variables: age and years of education).

	**L.SFG**	**L.IFGtri**	**L.STG**	**L.ITG**	**L. FG**	**Speech**	**Nouns**	**Verbs**
	**area**	**area**	**area**	**thickness**	**volume**	**rate**	**frequency**	**frequency**
**Males**								
L.SFG area	–	0.295	0.251	–0.223	0.178	0.333	–0.119	0.141
L.IFGtri area	0.295	–	0.366	–0.026	0.121	–0.184	–0.050	–0.385
L.STG area	0.251	0.366	–	−0.425*	0.598**	0.253	–0.046	–0.322
L.ITG thickness	–0.223	–0.026	−0.425*	–	0.064	–0.117	0.140	–0.175
L. FG volume	0.178	0.121	0.598**	0.064	–	0.206	0.051	–0.162
Speech rate	0.333	–0.184	0.253	–0.117	0.206	–	–0.230	0.340
Nouns frequency	–0.119	–0.050	–0.046	0.140	0.051	–0.230	–	–0.236
Verbs frequency	0.141	–0.385	–0.322	–0.175	–0.162	0.340	–0.236	–
**Females**								
L.SFG area	–	0.243	–0.072	0.070	–0.340	0.508**	0.163	–0.220
L.IFGtri area	0.243	–	0.116	0.290	–0.141	0.328	–0.145	0.060
L.STG area	–0.072	0.116	–	−0.421*	0.553**	–0.031	0.240	0.012
L.ITG thickness	0.070	0.290	0.421*	–	0.250	0.213	0.099	0.081
L. FG volume	–0.340	–0.141	0.553**	0.250	–	–0.275	0.331	–0.147
Speech rate	0.508**	0.328	–0.031	0.213	–0.275	–	–0.350	0.049
Nouns frequency	0.163	–0.145	0.240	0.099	0.331	–0.350	–	−0.528**
Verbs frequency	–0.220	0.060	0.012	0.081	–0.147	0.049	−0.528**	–

Significance levels: <0.01**, <0.05*.

For males, the basic model predicting speech rate from age and education was not significant [*F*(2,24) = 0.485, *p* = 0.622]. The more complex model predicting speech rate from demographic and anatomical variables also failed to reach significance [*F*(3,23) = 1.307, *p* = 0.296]. Neither model contained significant independent predictors. Furthermore, the additional variable explained by the inclusion of the SA metrics was not significant (*R*^2^ change = 0.034, *p* = 0.103).

Similarly, for females, the basic model predicting speech rate from demographic variables was not significant [*F*(2,27) = 0.828, *p* = 0.448]. In contrast to men, the multivariate complex model was significant for women [*F*(3,26) = 3.732, *p* = 0.024]. Within this model, older age (β = 0.419, *t* = 2.145, *p* = 0.042) and greater SFG SA (β = 0.554, *t* = 3.008, *p* = 0.006) were significantly associated with higher speech rate when model variables (including age and years of education and) were held constant. The between-model ANOVA revealed that the more complex model provided a significantly better prediction of females’ speech rate than demographic variables alone (*R*^2^ change = 0.243, *p* = 0.006).

Even though there were no significant differences between males and females with regard to DTI indices, we ran analyses with speech variables that were found to differ between sexes and anatomical metrics related to specific association fibers which support cortico-cortical connections within the perisylvian network. We therefore restricted our analyses to the left and right arcuate and inferior longitudinal fasciculi FA values (based on the available tracts of JHA). For each sex, we first conducted a principal component analysis on FA values extracted from all 100 bins for each tract. Please see Supplementary Table S1, for mean FA values from 100 bins per participant.

A series of multivariate regression models were conducted separately for men and women. In each model, the dependent variable was a language metric (i.e., speech rate, nouns, and verbs), and the independent variables included the retained principal components for each respective tract and nuisance regressors of age and years of education. Correction for multiple tests was performed using Benjamini and Hochberg’s (1995) false discovery rate (*p* < 0.05). None of the models were statistically significant.

## Discussion

To our knowledge this is the first study aiming to investigate sex differences in connected speech tasks in association with gray and white matter structures. Our results indicate that females present higher speech rate and verb frequency, while males have significantly higher noun frequency. No significant difference is apparent for number of utterances, MLUs and frequency of function words. Whole brain analysis of structural imaging data reveal differences in several clusters with regard to cortical thickness, surface area and gray matter volume, with men presenting higher values in all gray matter metrics in both hemispheres. In relation to white matter tracts, no significant differences appear in FA, MD, RD, and AD values of twenty-two tracts bilaterally (corpus callosum minor and major fibers, arcuate fasciculus, frontal aslant tract, superior and inferior longitudinal fasciculus, occipitofrontal fasciculus and uncinate fasciculus) on the basis of voxel-based analysis (tract based spatial statistics) and automated tractography. Regression analyses show that the superior frontal gyrus cluster may serve as a significant predictor of speech rate variance only in females.

### Speech Variables

Our results on sex differences in speech metrics seem compatible with previously reported research evidence. Wardle et al. (2011) showed that females presented higher speech rate compared to males on two free narration tasks, monolog and interpersonal speech, while there were no differences in total number of words and narration duration. Our data reveal comparable results for a similar task that refers to a personal narration. It should be although noted that our sample’s age range is wider than Wardle et al. (2011) who included younger participants of a narrower age range (18–35 years old) in their study. On the other hand, Ardila and Rosselli (1996) concluded that sex differences are apparent in total number of words produced, during a picture description task, with women being superior to men; however, the effect of sex was mediated by age in that case, i.e., women were found to utter significantly more words, but only for the age group of 51–65 years old. In an attempt to interpret their findings, the authors formulated the hypothesis that language alterations in older adults are sex-dependent, with the detrimental effect of age on speech output being prominent exclusively in males. This perspective seems to be further supported by Leeper and Culatta (1995) who found that older men presented a trend – yet statistically insignificant- for elevated frequency of dysfluencies in both a picture description and a free monolog task. On the other hand, when the task does not involve spontaneous speech but is limited to story retelling, no sex differences are noted in terms of the amount of produced words or the rate of speech (Juncos-Rabadán, 1996). The only study that showed male superiority in rate of speech output in free narration was the one conducted by Verhoeven et al. (2004), who found that Dutch- speaking males have higher speech rate than females in free monologs including general topics such as current affairs, education, hobbies and holidays. Evidence in favor of sex differences in language production regarding word classes and lexical richness are scarce. There are studies, which failed to find any discrepancies between males and females in word classes of nouns, verbs, adjectives, and connectors (e.g., Ardila and Rosselli, 1996). However, Singh’s (2001) findings for conversational speech of older healthy participants (above 50 years old) are in accordance with our results regarding sex differences in word class use. In that study, men were shown to use significantly more nouns, while the opposite trend appears for verbs. Despite the possible importance of this finding, the author attributes the aforementioned sex differences to a hypothesized differentiation in the organization of language between males and females without providing any further interpretation.

In sum, the lack of consensus regarding sex differences in connected speech is evident. This could be due to several factors, but one that should be highlighted is the adoption of different methodological approaches. There are several ways of spontaneous speech analysis suggested in the literature, which in turn may provide the experimenter with various linguistic indices, such as total number of words, words per minute, frequency of specific word classes, lexical richness, frequency of disfluencies or coherence. Nevertheless, it is practically impossible to include all these speech metrics in one single study, and therefore the task of encapsulating the complexity of spontaneous speech becomes utopic. In our study for example, we included several speech fluency metrics, as total number of words and narration duration, MLUs, words per minute and several words classes (nouns, verbs, and function words), similar to Ardila and Rosselli (1996) study, in an attempt to present an integral analysis of narration’s structural organization; however we didn’t include any dysfluency measures as Leeper and Culatta (1995) did.

In addition, we also need to acknowledge that studies aiming to investigate sex differences have used different speech genres. For example, Ardila and Rosselli (1996) utilized a well-known picture description task (cookie theft picture), while Wardle et al. (2011) focused on different aspects of free narration. It has been well established that different elicitation techniques may affect the qualitative and quantitative characteristics of the acquired speech sample, in the sense that production of distinct narrative genres may pose different cognitive and linguistic demands (Ulatowska et al., 1990; Fergadiotis et al., 2011). Consequently, comparison and synthesis of findings across the available studies becomes quite challenging.

One aspect of the current study that should not be overlooked is the emotional component of the language task used (for a discussion on the importance of elicitation tasks requiring narration of emotionally infused information, see Efthymiopoulou et al., 2017). Previous social studies have emphasized the existence of sex differences regarding expression of emotion (see Golombok and Fivush, 1994, for a review). More specifically, women appear to have a greater tendency to express emotions and describe emotional situations more frequently using verbal and non-verbal means (e.g., facial expressions) than men (see Brody and Hall, 1993, for a review). Fivush et al. (2000) found that mothers talked more about personal experiences, related for example to their children, compared to men. In addition, it has been shown that female adolescents tend to produce higher amount of speech during personal narrations with enhanced emotional component (Fivush et al., 2012). This notion is also related with some research evidence indicating sex differences in preference of the conversation topic. Ferber (1995) noted that on most occasions, men generally prefer to discuss about technical issues compared to women. Most studies fail to clarify the exact reasons why these differences emerge, referring to the interaction of biological differences and socio-cultural effects during development (Fivush et al., 2000; Wodak, 2015).

### Neuroimaging Data

Our study revealed several sex differences in terms of surface area, cortical thickness and gray matter volume bilaterally, with men presenting higher values in all cases. Our results are in agreement with most of the studies regarding surface area and gray matter volume (see for example Raz et al., 1997; Goldstein et al., 2001; Carne et al., 2006). Based on the large-scale study of 5126 participants conducted by Ritchie et al. (2018) males presented higher raw values of gray matter volume and surface area, including extensive bilateral brain regions, while females had higher raw thickness values mostly in left occipital and bilateral parietal cortices. In contrast, some studies suggest that women exhibit larger volume in several brain areas, such as the cingulate, the calcarine sulci, and the parahippocampal gyri (Good et al., 2001), as well as the right inferior parietal lobule (Chen et al., 2007). It should be noted that Ritchie et al. (2018) have used mean thickness as a control variable in their analysis for cortical thickness; however we decided not to follow this methodology as there is strong evidence that this practice could be proved misleading, especially for smaller samples (see Westman et al., 2013).^3^

Our white matter tract analysis revealed no significant differences between sexes in either voxel-based analysis using TBSS or tractography implemented with AFQ. Again, results derived from previous studies are controversial, as in some cases women seem to have higher fractional anisotropy values compared to men (Kanaan et al., 2012; Dunst et al., 2014; Kanaan et al., 2014), while others find the reverse pattern. More specifically, Ritchie et al. (2018) found the opposite result for eighteen tracts bilaterally, with males presenting higher FA values, and Inano et al. (2011) found that men present significantly higher FA values in regions including the splenium of the corpus callosum, posterior internal capsule, external capsule, cingulum and superior longitudinal fasciculus bilaterally, while women are shown to be superior only in the column of the fornix. The lack of significant sex differences in white matter tracts in our study could be possibly attributed to small sample size. As Ritchie et al. (2018) stated, larger samples may increase sensitivity with regard to the identification of sex effects on white matter structure. Despite the fact that our analyses did not yield significant results, large-scale studies suggest that sex should be included as a covariate when investigating structural, white matter-related variables.

### Speculation on Possible Sex-Dependent Patterns of Associations Between Structural Indices and Language Variables

Overall, our results show clear-cut differences between males and females with regard to language metrics and indices of gray matter volume, cortical area, and cortical thickness. However, sex-discrepancies appear to be evident only when we separately examine either brain structure or linguistic behavior. The sought-after integration of anatomical and language variables is far from clear. In particular, our data provide a single significant result toward the notion that any anatomical differences could reflect differences with regard to language indices and vice versa. The superior frontal gyrus area cluster seems to be a significant predictor of speech rate, but its predictive value is restricted to females. We will attempt to speculate on the meaning of this finding, keeping in mind that a significant relationship between a structural variable such as surface area and a rather complex language index such as speech rate, does not necessarily correspond to a functional association, and in any case these findings should be interpreted with caution. Notwithstanding these caveats, the first explanation of the regression results could be attributed to statistical factors. Simply put, variance is greater in women for both speech rate and superior frontal gyrus cluster surface area, compared to men. Greater variance could have allowed the emergence of association patterns only in women, in contrast to men, whose values do show much higher degree of skewedness, as well as a relatively restricted spreading around the central tendency index. Another possible explanation could be associated with the notion that greater area reflects greater computational capacity. This explanation makes sense given that females whose superior frontal gyrus surface area was at the higher end of the observed range also demonstrated higher speech rate. Brain regions included in the superior frontal gyrus cluster have been shown to be involved in higher executive functions, which could be involved in processing speed, selective retrieval and may be related to required cognitive resources for selection, retrieval, and combination of individual components of speech, such as semantic, phonological, and syntactic aspects (Petrides and Pandya, 2008). In other words, prefrontal cortices could be involved in semantic integration and organization of each utterance. This notion is further supported by brain imaging data indicating the involvement of the superior frontal gyrus in semantic processing, as a component of a network of frontal cortical regions, which is argued to facilitate domain-general semantic control (for a review see Price, 2010, 2012). A number of studies have shown that the mid dorsolateral prefrontal cortex, i.e., area 46 and 9/46v are involved in the monitoring of information within working memory (Champod and Petrides, 2007, 2010). Based on monkey anatomical studies, the aforementioned specific component of that prefrontal system is anatomically connected via Superior Longitudinal Fasciculus III with the homolog of the supramarginal gyrus, the ventral premotor cortex and area 44 (pars opercularis). It is therefore reasonable to assume that the mid dorsolateral monitoring system may be specialized for the monitoring of speech production/articulation within working memory (for a review on the role of mid dorsolateral prefrontal cortex in hierarchical control of behavior, see Badre and Nee, 2018; and in working memory, see Petrides, 2015). Therefore, smaller surface area in that region could reflect limited computational capacity, which could eventually result in slow speech rate. The above explanation should be further tested in order to acquire robust support.

An alternative explanation could be that the relationships between brain anatomy and speech variables are mediated by brain function, particularly considering the available evidence on different activation patterns during language tasks between males and females (e.g., Shaywitz et al., 1995; Jaeger et al., 1998; Kansaku et al., 2000; but see also Kaiser et al., 2009). If this is the case, then the first scenario proposed above (that any associations are due to the differential distribution of both behavioral and anatomical variables in males and females) could easily explain the fact that the statistically significant relationship between surface area and speech rate was evident only for women. The above explanation should be tested in future multimodal investigations regarding the existence of brain-based sex differences in language processing.

## Conclusion

Our study shows differences between sexes with regard to specific indices of narrative ability, and further illustrates their association with inter-sex differences in gray matter. Although there are several studies focusing on differences between healthy males and females regarding either brain structure or narrative skills, the literature lacks studies attempting to integrate the observed discrepancies between the two sexes. We therefore argue that our findings contribute to the understanding of the differences in linguistic behavior and brain structure, as well as the association between the former and the latter, and further stress the need for future research on this field with larger cohorts and the inclusion of other factors (e.g., demographic or developmental) or multiple assessments with a range of speech samples in order to reassure test retest stability, that possibly affect language or structural variables, differentially between men and women.

## Data Availability Statement

The datasets generated for this study will not be made publicly available. Special requests should be made to both the Neurology and Radiology Departments of the National and Kapodistrian University of Athens, by contacting CP, cpotagas@otenet.gr.

## Ethics Statement

The studies involving human participants were reviewed and approved by Eginition Hospital in Athens, School of Medicine, Greece. The patients/participants provided their written informed consent to participate in this study.

## Author Contributions

GA, EM, and DK conceived and designed the study, analyzed behavioral and neuroimaging data, and wrote the manuscript. YP performed the neuroimaging data analysis and revised the manuscript. DT performed neuropsychological testing, contributed to behavioral data analysis, and revised the manuscript. GV and EK designed the imaging acquisitions protocol, acquired the neuroimaging data, and revised the manuscript. NK supervised the neuroimaging data collection process and revised the manuscript. DG contributed to study conception and design and revised the manuscript. CP conceived and designed the study, supervised the behavioral data collection process, and revised the manuscript. SK conceived and designed the study and revised the manuscript.

## Conflict of Interest

SK is a scientific consultant for The Learning Corporation, but there is no overlap between this role and the submitted investigation. The remaining authors declare that the research was conducted in the absence of any commercial or financial relationships that could be construed as a potential conflict of interest.

## Footnotes

1 https://surfer.nmr.mgh.harvard.edu

2 http://www.fmrib.ox.ac.uk/fsl

3 We did run an analysis including mean thickness as a nuisance variable and the results were similar, i.e., we found higher cortical thickness for women. However, due to the methodological issue mentioned in the text, this analysis is not reported in the paper.
