# Supplementary material for: Investigating Gray and White Matter Structural Substrates of Sex Differences in the Narrative Abilities of Healthy Adults
Source: Front Neurosci. 2020 Jan 29;13:1424. doi: 10.3389/fnins.2019.01424 (PMC7000661; doi:10.3389/fnins.2019.01424)
Supplement: Supplementary file 1 [file Table_1.docx]

**Supplementary Table**. Individual fractional anisotropy (FA) values per tract for males and females’ groups, derived from Automated Fiber Quantification.

Males

| Participants | Age | Corpus Callosum | | Left Hemisphere | | | | Right Hemisphere | | | |
| --- | --- | --- | --- | --- | --- | --- | --- | --- | --- | --- | --- |
|  |  | Major | Minor | Arcuate | IFOF | Uncinate | ILF | Arcuate | IFOF | Uncinate | ILF |
| 1 | 60.00 | 0.6493 | 0.4858 | 0.5159 | 0.4591 | 0.4596 | 0.4670 | 0.4308 | 0.4871 | 0.4358 | 0.4421 |
| 2 | 64.00 | 0.5499 | 0.4657 | 0.4921 | 0.4884 | 0.3875 | 0.4405 | 0.4676 | 0.4779 | 0.3915 | 0.4319 |
| 3 | 51.00 | 0.6500 | 0.5220 | 0.4887 | 0.5044 | 0.5266 | 0.4477 | 0.4259 | 0.4983 | 0.4625 | 0.4071 |
| 4 | 36.00 | 0.6109 | 0.5370 | 0.4944 | 0.4609 | 0.4300 | 0.4681 | 0.4426 | 0.4606 | 0.3923 | 0.4450 |
| 5 | 53.00 | 0.6463 | 0.5206 | 0.5070 | 0.4735 | 0.4572 | 0.4681 | 0.4009 | 0.4635 | 0.4061 | 0.3793 |
| 6 | 51.00 | 0.6440 | 0.5094 | 0.4878 | 0.5055 | 0.4338 | 0.4420 | 0.4191 | 0.4790 | 0.3925 | 0.4337 |
| 7 | 40.00 | 0.6167 | 0.5140 | 0.4980 | 0.4847 | 0.4768 | 0.4319 | 0.4559 | 0.4969 | 0.4457 | 0.3786 |
| 8 | 38.00 | 0.6561 | 0.5673 | 0.4841 | 0.5279 | 0.4293 | 0.4607 | 0.4370 | 0.5481 | 0.4404 | 0.4500 |
| 9 | 51.00 | 0.6589 | 0.5054 | 0.4875 | 0.4609 | 0.4589 | 0.4505 | 0.3677 | 0.4517 | 0.4318 | 0.4280 |
| 10 | 40.00 | 0.6228 | 0.5257 | 0.5150 | 0.4936 | 0.5117 | 0.4400 | 0.4351 | 0.4719 | 0.4342 | 0.4313 |
| 11 | 40.00 | 0.6118 | 0.5427 | 0.5454 | 0.5127 | 0.4260 | 0.4555 | 0.4794 | 0.4792 | 0.3997 | 0.4176 |
| 12 | 48.00 | 0.6570 | 0.5419 | 0.5322 | 0.5527 | 0.4934 | 0.4553 | 0.5431 | 0.5041 | 0.4431 | 0.3819 |
| 13 | 30.00 | 0.6722 | 0.5090 | 0.5147 | 0.5210 | 0.4192 | 0.4423 | N/A | 0.5165 | 0.4356 | 0.4403 |
| 14 | 38.00 | 0.6370 | 0.5386 | 0.5184 | 0.5311 | 0.4185 | 0.4513 | 0.4580 | 0.5210 | 0.4243 | 0.4454 |
| 15 | 39.00 | 0.6640 | 0.5776 | 0.5050 | 0.5219 | 0.4263 | 0.5012 | 0.4059 | 0.5074 | 0.4146 | 0.4671 |
| 16 | 60.00 | 0.6228 | 0.4678 | 0.4986 | 0.4914 | 0.4667 | 0.4438 | 0.3915 | 0.4978 | 0.4177 | 0.4332 |
| 17 | 40.00 | 0.6403 | 0.5529 | 0.5127 | 0.5191 | 0.5165 | 0.4918 | 0.4495 | 0.5424 | 0.4503 | 0.4388 |
| 18 | 61.00 | 0.5976 | 0.5040 | 0.5106 | 0.4868 | 0.4285 | 0.4024 | 0.4089 | 0.4815 | 0.4083 | 0.3794 |
| 19 | 25.00 | 0.5886 | 0.5430 | 0.5144 | 0.4987 | 0.4881 | 0.4308 | 0.4831 | 0.5340 | 0.4711 | 0.4386 |
| 20 | 59.00 | 0.5525 | 0.5245 | 0.5216 | 0.5245 | 0.4843 | 0.4725 | 0.4783 | 0.4968 | 0.4920 | 0.4236 |
| 21 | 22.00 | 0.6076 | 0.5629 | 0.4817 | 0.5112 | 0.4721 | 0.4700 | 0.3911 | 0.5100 | 0.4493 | 0.4547 |
| 22 | 64.00 | 0.5968 | 0.5478 | 0.5573 | 0.5150 | 0.4839 | 0.4460 | 0.4963 | 0.4910 | 0.4885 | 0.4591 |
| 23 | 19.00 | 0.5955 | 0.5250 | 0.5674 | 0.5219 | 0.4667 | 0.5089 | 0.4977 | 0.5416 | 0.4462 | 0.4908 |
| 24 | 26.00 | 0.6814 | 0.5643 | 0.5261 | 0.5404 | 0.4291 | 0.4652 | 0.4286 | 0.5466 | 0.3944 | 0.4633 |
| 25 | 51.00 | 0.6749 | 0.4823 | 0.5149 | 0.4913 | 0.4906 | 0.4794 | 0.5399 | 0.4870 | 0.4528 | 0.4483 |
| 26 | 25.00 | 0.6324 | 0.5807 | 0.4786 | 0.5222 | 0.4705 | 0.4639 | 0.4583 | 0.5175 | 0.4428 | 0.4667 |
|  |  |  |  |  |  |  |  |  |  |  |  |
|  |  |  |  |  |  |  |  |  |  |  |  |

Females

| Participants | Age | Corpus Callosum | | Left Hemisphere | | | | Right Hemisphere | | | |
| --- | --- | --- | --- | --- | --- | --- | --- | --- | --- | --- | --- |
|  |  | Major | Minor | Arcuate | IFOF | Uncinate | ILF | Arcuate | IFOF | Uncinate | ILF |
| 1 | 65.00 | 0.6175 | 0.4706 | 0.4967 | 0.5125 | 0.3982 | 0.4673 | 0.4568 | 0.4929 | 0.4490 | 0.4086 |
| 2 | 57.00 | 0.6868 | 0.5345 | 0.4646 | 0.5154 | 0.4791 | 0.4701 | 0.3854 | 0.5283 | 0.4270 | 0.4314 |
| 3 | 50.00 | 0.6450 | 0.4902 | 0.4811 | 0.4811 | 0.4504 | 0.4609 | 0.4568 | 0.4322 | 0.4240 | 0.3994 |
| 4 | 48.00 | 0.6077 | 0.5358 | 0.5090 | 0.5172 | 0.4897 | 0.4546 | 0.4947 | 0.4996 | 0.4103 | 0.4160 |
| 5 | 57.00 | 0.5411 | 0.5098 | 0.5206 | 0.4652 | 0.4560 | 0.4601 | 0.4607 | 0.4487 | 0.4399 | 0.4393 |
| 6 | 30.00 | 0.6107 | 0.5784 | 0.5396 | 0.5428 | 0.4716 | 0.4921 | 0.4859 | 0.5257 | 0.4129 | 0.4348 |
| 7 | 36.00 | 0.6382 | 0.5552 | 0.5300 | 0.5064 | 0.4768 | 0.5124 | 0.5143 | 0.4551 | 0.3957 | 0.4398 |
| 8 | 50.00 | 0.5762 | 0.4923 | 0.5214 | 0.5353 | 0.4171 | 0.4376 | 0.4655 | 0.4860 | 0.3965 | 0.3863 |
| 9 | 60.00 | 0.6781 | 0.5822 | 0.5184 | 0.5356 | 0.4732 | 0.4382 | 0.4121 | 0.5019 | 0.4160 | 0.4018 |
| 10 | 35.00 | 0.5670 | 0.4962 | 0.4684 | 0.4651 | 0.4478 | 0.4218 | 0.4524 | 0.4753 | 0.4149 | 0.3836 |
| 11 | 51.00 | 0.5332 | 0.5491 | 0.5005 | 0.5086 | 0.4600 | 0.4824 | N/A | 0.5028 | 0.4457 | 0.4831 |
| 12 | 53.00 | 0.5590 | 0.5244 | 0.4760 | 0.4485 | 0.4180 | 0.4254 | N/A | 0.4504 | 0.3928 | 0.3909 |
| 13 | 52.00 | 0.6276 | 0.5267 | 0.5344 | 0.4733 | 0.4690 | 0.4592 | 0.5027 | 0.4883 | 0.4221 | 0.4303 |
| 14 | 38.00 | 0.5975 | 0.5755 | 0.5182 | 0.4957 | 0.4169 | 0.4504 | 0.4233 | 0.5086 | 0.3809 | 0.4691 |
| 15 | 41.00 | 0.6266 | 0.5528 | 0.5145 | 0.5290 | 0.4877 | 0.5151 | 0.4500 | 0.5249 | 0.4598 | 0.4468 |
| 16 | 42.00 | 0.6338 | 0.4949 | 0.5329 | 0.5066 | 0.4813 | 0.4466 | 0.4664 | 0.4807 | 0.4418 | 0.4536 |
| 17 | 42.00 | 0.6204 | 0.4900 | 0.5195 | 0.4731 | 0.4176 | 0.5075 | 0.4434 | 0.4598 | 0.3910 | 0.4791 |
| 18 | 45.00 | 0.6531 | 0.5152 | 0.5451 | 0.4918 | 0.5194 | 0.4529 | 0.5284 | 0.5000 | 0.4428 | 0.4293 |
| 19 | 45.00 | 0.6470 | 0.5277 | 0.5181 | 0.4864 | 0.4629 | 0.4569 | 0.4311 | 0.4998 | 0.4354 | 0.4211 |
| 20 | 53.00 | 0.6200 | 0.4918 | 0.5232 | 0.5054 | 0.4391 | 0.4375 | 0.5602 | 0.4947 | 0.3913 | 0.4312 |
| 21 | 23.00 | 0.6380 | 0.5558 | 0.5268 | 0.5108 | 0.4475 | 0.4626 | 0.4772 | 0.5038 | 0.3980 | 0.4687 |
| 22 | 56.00 | 0.5746 | 0.5108 | 0.5019 | 0.5006 | 0.4455 | 0.4851 | 0.4910 | 0.5020 | 0.4616 | 0.4650 |
| 23 | 49.00 | 0.6660 | 0.5545 | 0.5745 | 0.5107 | 0.4793 | 0.4486 | 0.4822 | 0.4872 | 0.4510 | 0.4268 |
| 24 | 53.00 | 0.6279 | 0.4836 | 0.4880 | 0.4625 | 0.4290 | 0.4774 | 0.4288 | 0.5108 | 0.4470 | 0.4569 |
| 25 | 37.00 | 0.6458 | 0.5385 | 0.4977 | 0.4858 | 0.4403 | 0.4558 | 0.4233 | 0.4434 | 0.3846 | 0.3929 |
| 26 | 37.00 | 0.6347 | 0.5639 | 0.5156 | 0.5618 | 0.5044 | 0.4460 | 0.4575 | 0.5622 | 0.4686 | 0.4515 |
| 27 | 29.00 | 0.6399 | 0.5564 | N/A | 0.5073 | 0.4668 | 0.4821 | 0.4891 | 0.5125 | 0.5002 | 0.4607 |
| 28 | 21.00 | 0.6088 | 0.5729 | 0.5232 | 0.4871 | 0.4715 | 0.4548 | 0.4877 | 0.4977 | 0.4600 | 0.4109 |
| 29 | 33.00 | 0.6793 | 0.6126 | 0.5414 | 0.5090 | 0.4599 | 0.4798 | 0.5245 | 0.5125 | 0.5000 | 0.4732 |
|  |  |  |  |  |  |  |  |  |  |  |  |
|  |  |  |  |  |  |  |  |  |  |  |  |
